# The Effectiveness of an eHealth Family-Based Intervention Program in Patients With Uncontrolled Type 2 Diabetes Mellitus (T2DM) in the Community Via WeChat: Randomized Controlled Trial

**DOI:** 10.2196/40420

**Published:** 2023-03-20

**Authors:** Yuheng Feng, Yuxi Zhao, Linqi Mao, Minmin Gu, Hong Yuan, Jun Lu, Qi Zhang, Qian Zhao, Xiaohong Li

**Affiliations:** 1 Department of Health Policy and Management School of Public Health Fudan University Shanghai China; 2 China Research Center on Disability Issues Fudan University Shanghai China; 3 Key Laboratory of Health Technology Assessment National Health Commission Fudan University Shanghai China; 4 Juyuan New District Community Health Service Center, Jiading District Shanghai China; 5 Shanghai Jiading District Center for Disease Control and Prevention Shanghai China; 6 School of Community and Environmental Health Old Dominion University Norfolk, VA United States

**Keywords:** public health, type 2 diabetes mellitus, intervention, randomized controlled trial, community health center

## Abstract

**Background:**

Intervention based on family support and risk perception can enhance type 2 diabetes mellitus (T2DM) patients’ self-care activities. In addition, eHealth education is considered to improve family members’ support for patients with T2DM. However, there is little evidence from rigorously designed studies on the effectiveness of an intervention combining these approaches.

**Objective:**

This randomized controlled trial (RCT) aimed to assess the effectiveness of an eHealth family-based health education intervention for patients with T2DM to improve their glucose control, risk perception, and self-care behaviors.

**Methods:**

This single-center, 2-parallel-group RCT was conducted between 2019 and 2020. Overall, 228 patients were recruited from Jiading District, Shanghai, and randomly divided into intervention and control groups. The intervention group received an eHealth family intervention based on community management via WeChat, whereas the control group received usual care. The primary outcome was the glycated hemoglobin (HbA_1c_) level of the patients with T2DM, and the secondary outcomes were self-management behavior (general and specific diet, exercise, blood sugar testing, foot care, and smoking), risk perception (risk knowledge, personal control, worry, optimism bias, and personal risk), and family support (supportive and nonsupportive behaviors). A 2-tailed paired-sample *t* test was used to compare the participants at baseline and follow-up within the control and intervention groups. An analysis of covariance was used to measure the intervention effect.

**Results:**

In total, 225 patients with T2DM were followed up for 1 year. After intervention, they had significantly lower HbA_1c_ values (β=–.69, 95% CI –0.99 to –0.39; *P*<.001). They also had improved general diet (β=.60, 95% CI 0.20 to 1.00; *P*=.003), special diet (β=.71, 95% CI 0.34 to 1.09; *P*<.001), blood sugar testing (β=.50, 95% CI 0.02 to 0.98; *P*=.04), foot care (β=1.82, 95% CI 1.23 to 2.42; *P*<.001), risk knowledge (β=.89, 95% CI 0.55 to 1.24; *P*<.001), personal control (β=.22, 95% CI 0.12 to 0.32; *P*<.001), worry (β=.24, 95% CI 0.10 to 0.39; *P*=.001), optimism bias (β=.26, 95% CI 0.09 to 0.43; *P*=.003), and supportive behaviors (β=5.52, 95% CI 4.03 to 7.01; *P*<.001).

**Conclusions:**

The eHealth family-based intervention improved glucose control and self-care activities among patients with T2DM by aiding the implementation of interventions to improve T2DM risk perceptions among family members. The intervention is generalizable for patients with T2DM using health management systems in community health centers.

**Trial Registration:**

Chinese Clinical Trial Registry ChiCTR1900020736; https://www.chictr.org.cn/showprojen.aspx?proj=31214

## Introduction

### Background

Worldwide, type 2 diabetes mellitus (T2DM) has become a serious public health problem; it leads to severe health outcomes and creates a heavy economic burden [1,2]. The prevalence of T2DM is rising across all regions [1]. For people aged over 60 years, the prevalence of T2DM exceeds 20%, and it is expected to increase with the aging of the world’s population [1].

China has the highest number of people with diabetes and mortality globally [1]. Among all types of diabetes, T2DM accounts for over 90% of diagnoses, and this type of the disease can cause various complications [3]. In 2021, health expenditures in China related to T2DM and complications reached about US $0.17 trillion, accounting for 1% of gross domestic product [1,4]. Therefore, it is urgent to explore practical measures to reduce the occurrence of T2DM and prevent complications from occurring in patients with T2DM.

Poor glucose control is associated with the occurrence of complications [5]. Glycated hemoglobin (HbA_1c_) can be used to determine glucose control level [6]. HbA_1c_ ≥7% indicates that a patient has poor glucose control [7].

If HbA_1c_ increases by 2%, the risk of T2DM all-cause mortality increases by 40% to 86% in the following 10 to 20 years [8]. However, most Chinese patients with T2DM have poor glucose control [9]. Studies show that many factors influence glucose control, such as psychosocial factors [10], self-care activities [11], risk perception [12], and social support [13].

China released the National Standard for Basic Public Health Services in 2009 [14], stating that the health management of T2DM is one of the main areas of focus and that community health service centers should regularly provide usual care to patients with T2DM, such as face-to-face follow-up, health education, physical examination, and glucose testing. Until 2016, although a large number of patients with T2DM received standardized management at community health service centers [15,16], the effectiveness of usual care was not good, leading to a failure to enhance perceptions among patients with T2DM of the importance of glucose control and improving self-care activities. The effectiveness of usual care is thus unsustainable due to poor understanding and compliance with such aspects of care as regular face-to-face follow-ups and glucose testing [17]. Therefore, it is essential to explore an effective way to implement health education interventions for patients with T2DM at the community level.

### Prior Studies

Prior studies have explored various ways to improve self-care activities among patients with T2DM and improve interventions in many aspects.

First, the effectiveness of smartphone-based interventions has been confirmed for intervention platforms, and they can improve glucose control levels and self-care activities [18,19]. The development of technologies and apps that are self-developed or can be downloaded in app stores provides a valuable intervention tool for patients with diabetes [20-22]. Kho et al [23] developed an empirical diabetes app, but developing and popularizing such interventions is expensive, and they are difficult to generalize. Therefore, it is vital to explore cheap and generalizable intervention platforms. In China, WeChat, a free and popular social media platform with about 576 million users as of 2017, is used by people in daily life, [24,25] providing a potential platform to develop a generalized intervention program.

Second, the content of the intervention must focus on self-care activities, because these are important to achieve therapeutic targets and prevent the development of complications [26]; many prior interventions have therefore focused on self-care activities [27,28]. A majority of interventions implemented health education on behaviors such as diet, exercise, and foot care [27]. Self-care activities are directly influenced by T2DM risk knowledge and attitudes, such as risk perception [29]. However, few studies have conducted interventions via comprehensive programs [30]. Exploring a comprehensive intervention program that targets knowledge, health beliefs, and self-care behaviors is essential to improve glucose control among patients with T2DM [29,30], help them participate in community health management, and improve their communication with doctors via family members.

Third, for intervention subjects, prior studies focused on patients with T2DM [31], peers [32], and family members [33]. Family is an essential social support resource for self-care activities among patients with T2DM [34]. However, implementation of family-based intervention is difficult due to low participation rates of family members [35].

### Aim

The study aimed to develop an eHealth family-based intervention program targeting knowledge, attitude, and behaviors and validate whether the intervention could improve glucose control among patients with T2DM registered in community health centers through a rigorously designed trial.

## Methods

### Study Design

According to the study protocol [36], the conceptual framework for this study was that the eHealth family-based intervention via WeChat would improve family members’ knowledge and risk perception of T2DM, which could help patients with T2DM to practice self-management, promote participation in community T2DM management, and improve communication with doctors, so that glucose control among patients with T2DM would ultimately be improved.

We conducted a single-center, 2-parallel-group randomized controlled trial that lasted 1 year to evaluate the effectiveness of this family-based intervention. Author LM was responsible for generating a random allocation sequence. The community health center enrolled the participants and assigned them to the intervention group or the control group. The intervention group received the eHealth intervention and usual care. The control group received usual care. The primary outcome was the HbA_1c_ value. The secondary outcomes were self-care activities, risk perception, and family support. Notably, because the intervention was an eHealth intervention, participants knew they would receive the intervention when they provided informed consent; thus, the study participants could not be blinded. The study design strictly followed the Consolidated Standards of Reporting Trials (CONSORT) eHealth checklist (version 1.6.1).

### Participants

According to the National Standard for Basic Public Health Services [14], community health service centers should maintain correspondence with patients and determine the prevalence of diabetes. This randomized control trial was conducted in the central area of Jiading District, which includes 2 community health service centers: Jiading Town Community Health Service Center and Juyuan New District Community Health Service Center. A total of 3874 individuals with diabetes were registered at these community health service centers. Of 1650 individuals with recorded HbA_1c_ values, 879 had a value over 7%.

The inclusion criteria for the patients were as follows: (1) they were registered in 1 of the 2 community health service centers in the urban area in Jiading District, (2) they had been diagnosed with type 2 diabetes by a doctor at least 6 months before study enrollment, (3) they were aged 18 to 79 years, (4) their HbA_1c_ level was ≥7%, (5) they had no plans to leave their place of residence in the following 12 months, and (6) they had a family member who could use WeChat and lived with the patient or visited them at least once a week.

The exclusion criteria for the patients were as follows: (1) they had other serious illnesses or illnesses not suitable for this study, (2) they were women who were pregnant or preparing for pregnancy, (3) they were unable to complete the 12-month follow-up for reasons such as moving and not transferring to another health care facility, (4) they were unwilling or unable to provide informed consent, and (5) they were currently participating in another intervention study.

Patients were required to choose 1 family member as a supporter to receive the corresponding intervention. The inclusion and exclusion criteria for the family members were as follows: (1) they were in regular contact with the patient, (2) they were nominated by the patient, (3) they were older than 18 years, and (4) they had never participated in other, similar research.

### Patients and Public Involvement

Patients and the public were not involved in our research’s design, conduct, reporting, or dissemination plans.

### Sample Size Calculation

We calculated the sample size based on a formula for comparing 2 means with a ratio of 1:1 between the intervention and control groups. This study used the HbA_1c_ value as the most important indicator. The formula for comparing 2 sample means was as follows:




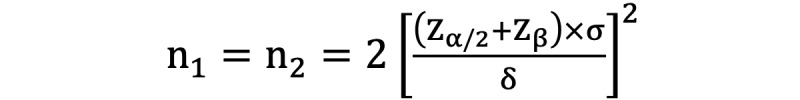




We set α=.05 and β=.20. δ was the effect size, which ranged from 0.25 to 0.70 [37-39]. In this study, we supposed that the effect size was 0.50, and σ (standard deviation) was 1.2 [36]. Therefore, about 91 participants were required in each group. Considering a dropout rate of 20%, we planned to recruit about 110 patients in each group. The ratio of family members to patients was 1:1.

### Intervention Tool

**Figure 1:**
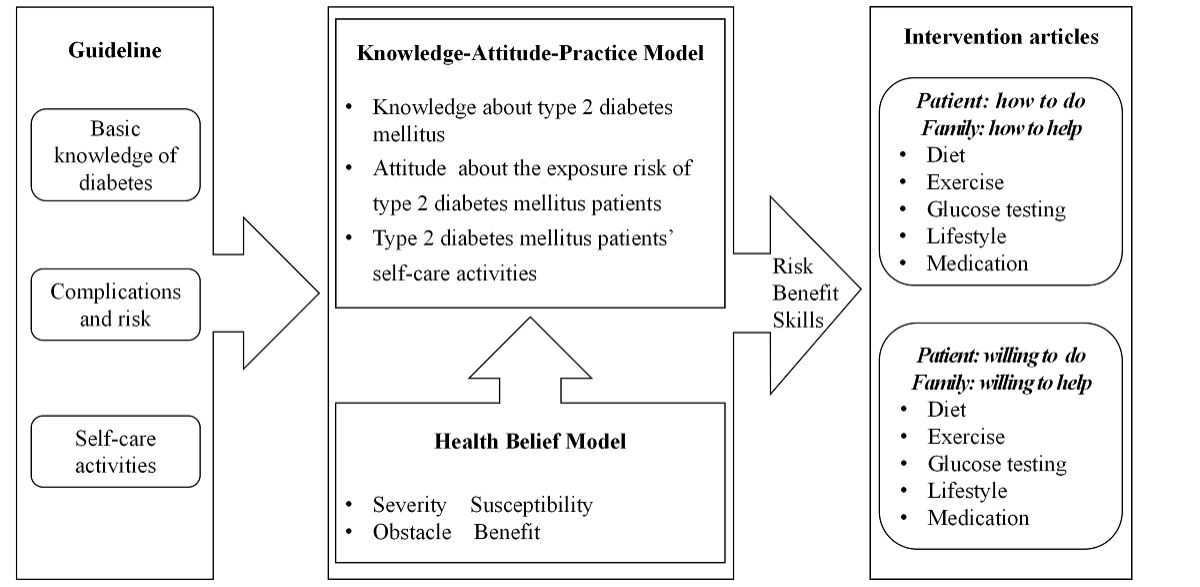
Design of the online intervention articles based on the Knowledge-Attitude-Practice model and the Health Belief Model.

The study established an official WeChat account called Jiading Sugar Steward, which included 3 modules: blood glucose data, complications, and notices. The intervention was implemented by using this account to deliver intervention articles. First, following the Guidelines for the Prevention and Treatment of T2DM, the study searched 4 domains of health intervention, including diabetes knowledge, complications, risk, and self-care activities (eg, diet, exercise, medication, lifestyle, glucose testing, and skills). All intervention articles were then developed based on the Knowledge-Attitude-Practice (KAP) model [40] and the Health Belief Model (HBM) [41]. The KAP model guided the relationship between basic knowledge and risk knowledge about T2DM, behaviors related to improving daily self-care activities among patients with T2DM, and family members’ attitudes on reminding patients with T2DM to improve their self-care activities. The HBM guided two aspects of family members’ attitudes: (1) knowledge that T2DM can induce significant health complications and (2) knowledge of the benefits of self-care activities and the risk of not performing these activities. We used the KAP model and the HBM to develop 38 comprehensive articles on topics including basic knowledge, skills, and risk knowledge, that is, behaviors and psychology (Figure 1 and Multimedia Appendix 1). All articles were divided into 3 categories: acquisition (A-level articles, scored 4 to 5), familiarity (B-level articles, scored 3), and understanding (C-level articles, scored 1 to 2).

### Intervention Process

The intervention was composed of an online intervention and an in-person intervention.

#### Online Intervention: Family Members

The online intervention implementation included 3 phases. During phase 1 (from January 1 to February 28, 2019), family members of patients with T2DM in the intervention group were asked to follow the aforementioned official WeChat account and add it to their personal WeChat. During phase 2 (from March 1 to May 31, 2019), the study regularly delivered the 38 articles described above (about 3 articles were delivered per week, on Monday, Wednesday, and Friday). A-level articles were sent one-to-one via the official WeChat account and messages. B-level articles were sent by forwarding individual WeChat Moments. C-level articles were released through the official WeChat account (Multimedia Appendix 2). All online intervention articles were delivered at 6 AM or 5 PM on the push day so that subjects could receive the intervention outside of working hours. During phase 3 (June 1, 2019, to February 29, 2020), we measured the effectiveness of the online intervention.

#### In-Person Intervention: Patients With T2DM

The National Standard for Basic Public Health Services [14] calls for all patients with T2DM to receive on-site health education once every 3 months. For the in-person intervention in this study, which we compared with usual care (diet control, glucose testing, and auxiliary activities during exercise), we developed risk-perception–based health education courses focused on the practice of skills. Taking exercise as an example, we conducted health education on how patients with T2DM should properly exercise to manage their glucose levels. We also educated patients with T2DM on the risks and benefits of self-care activities. An example of a risk is that patients with T2DM cannot spend a long time out of the home because of the possibility of their glucose becoming low. An example of a benefit is that exercise can help control glucose.

The intervention measure was implemented by family practitioners and doctors in the prevention and health section. Family practitioners were mainly responsible for conducting follow-ups every 3 months. Doctors in the prevention and health section were mainly responsible for background operations, maintaining the official WeChat account, and cooperating with family practitioners.

### Outcome Measures

#### Primary Outcome

HbA_1c_ has been clinically used as a gold standard for assessing long-term blood glucose control and reflects blood glucose concentration over approximately 3 months [42,43]. Therefore, we selected HbA_1c_ as the primary outcome. HbA_1c_ was measured at Jiading Central Hospital. A lower HbA_1c_ level means that a patient with T2DM has better glucose control.

#### Secondary Outcomes

Four secondary outcomes were measured by questionnaires that were confirmed to be suitable for use by Chinese people. The questionnaires covered topics including diabetes self-care activities [44], risk perception of diabetes [45], and family support [46].

### Self-care Activities

Self-care activities were measured using the Summary of Diabetes Self-Care Activities scale [47], which measures normal diet, abnormal diet, exercise, blood glucose monitoring, foot care, and smoking. Besides the last item, which is scored from 1 to 2, the other items range from 0 to 7. The higher the final score, the better the patient’s self-care behavior. A higher score means that a patient with T2DM has better performance of self-care activities.

### Risk Perception

Risk perception was examined using the Risk Perception Survey–Diabetes Mellitus scale [48], which is divided into 2 modules (risk perception and risk knowledge), with 31 entries in 6 dimensions. However, the dimension “comparative environmental risk” was removed because it is unsuitable for people with a Chinese cultural background. This decision was made after expert consultation and interviews with patients with T2DM and their family members. The meanings of the remaining 5 dimensions are shown in Multimedia Appendix 3. The total risk knowledge score is the sum of the 5 items.

### Family Support

Family support was evaluated using the Diabetes Family Behavior Checklist [49], which contains 16 items scored from 1 to 5. A score of 1 means that the patient never receives support from their family. A score of 5 means that the family always supports the patient. This indicator includes positive support and negative support. A higher positive support score or lower negative support score mean patients with T2DM have a better family living environment with better family support behaviors.

### Data Collection and Randomization

From January 1 to February 28, 2019, the study recruited participants according to the inclusion and exclusion criteria. Eventually, 228 pairs of participants were enrolled in each group. They were randomly allocated to the intervention and control groups at the individual level via a random-number table; 114 pairs of participants were assigned to the intervention and control groups. The intervention measure was then implemented for a period of 12 months.

To measure HbA_1c_, we collected blood samples at baseline and the 12th month. Laboratory tests were done at the Jiading Central Hospital. Secondary outcomes were collected via on-site questionnaire surveys at baseline and the 12th month. The baseline questionnaire survey was conducted by 4 authors (XL, LM, YZ, and YF), who collected data from patients with T2DM and their family members individually at the community health service centers. At the 12th month, the participants were contacted by telephone and the research team re-collected their data.

### Statistical Analysis

Sociodemographic characteristics were summarized for the intervention and control groups. Frequencies and percentages were used for categorical variables and mean (SD) for continuous variables.

To assess the effectiveness of the intervention, a 2-tailed paired-sample *t* test was used to compare the baseline and follow-up data in the intervention and control groups. Changes between the baseline and follow-up period in the intervention and control groups were measured with the 95% CI at baseline and the 12th month. Finally, since HbA_1c_ was the primary outcome, we further analyzed sex subgroups to explore whether the results differed between males and females. Furthermore, we used an analysis of covariance to clarify the intervention effect and included the sex, age, and education of patients with T2DM, the family members’ education, and the family members’ relationship as covariables.

All data management and analyses were performed using Stata (version 15.0; Stata Corp). We set α=.05 and β=.20; the power was 80%. Statistical significance was set at *P*<.05.

### Ethics Approval

Informed consent was provided by all participants. In addition, the trial was ethically approved by the Medical Research Ethics Committee of the School of Public Health Fudan University (2018-01-0663). All participants provided written informed consent.

## Results

### Participant Characteristics

**Figure 2:**
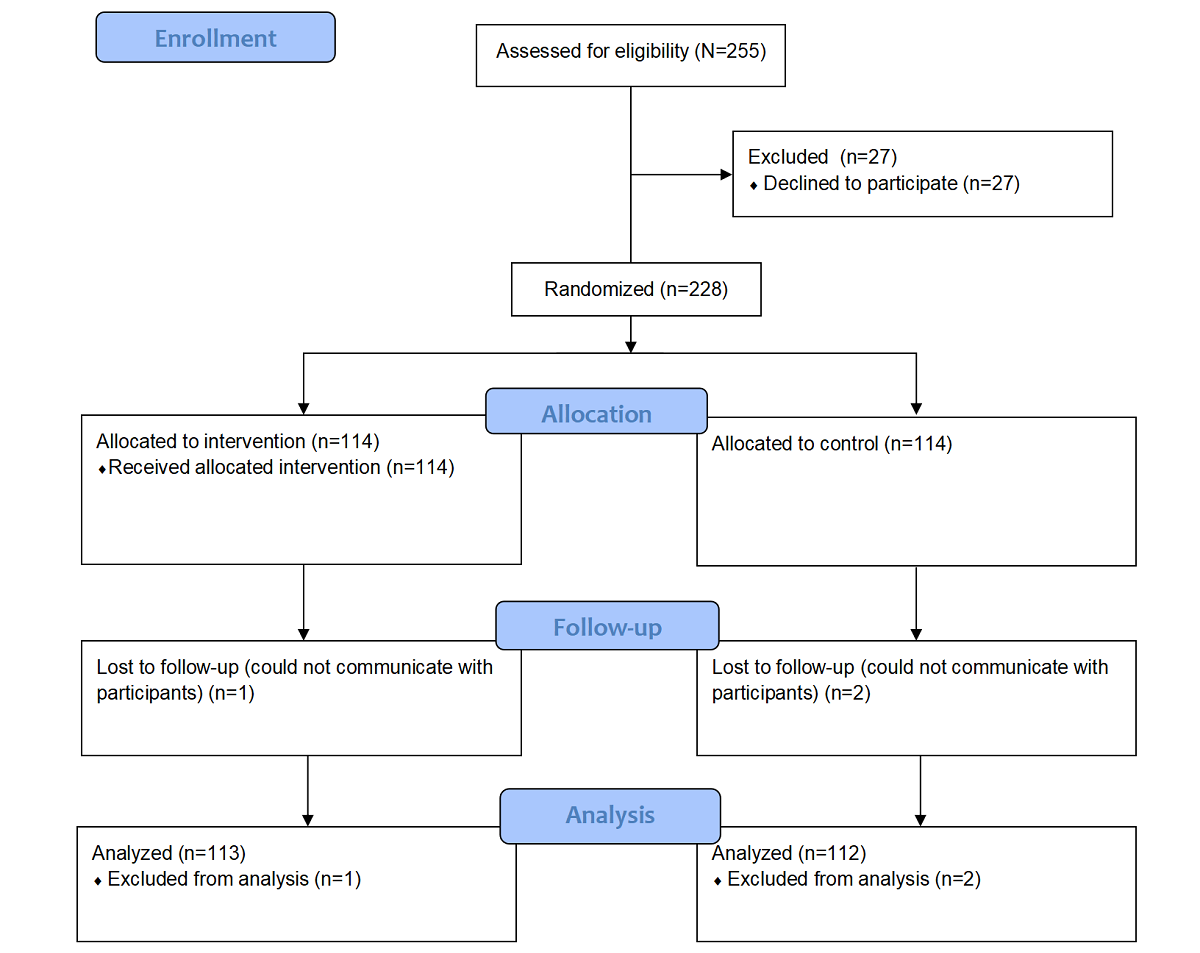
Flow diagram of trial participation.

In total, 225 patients (113 patients in the intervention group and 112 patients in the control group) completed this 1-year intervention study. Three participants (1 in the intervention group and 2 in the control group) were lost to follow-up. Figure 2 shows the process of inclusion in each analysis, starting with the originally assigned groups.

Among the 225 patients, 48.4% (n=109) were men and 51.6% (n=116) were women, with a mean age of 65.6 (SD 7.1) years. Of the 225 family members nominated by the included patients, 48% (n=108) were men and 52% (n=117) were women, with a mean age of 48.5 years. Spouses were the family members who most commonly lived with the patients. The baseline sociodemographic and clinical characteristics of the patients and their family members are shown in Table 1.

**Table 1:** Characteristics of the patients and their family members at baseline.

Participants/characteristics				Intervention group (n=113)		Control group (n=112)		*P* value	
**Patients with type 2 diabetes**									
	**Gender, n (%)**								.64
		Male	53 (46.9)		56 (50)				
		Female	60 (53.1)		56 (50)				
	Age (years), mean (SD)			65.7 (6.7)		65.4 (7.5)		.78	
	**Marriage status, n (%)**								.20
		Married	106 (93.8)		98 (87.5)				
		Divorced	2 (1.8)		2 (1.8)				
		Widowed	5 (4.4)		12 (10.7)				
	**Education, n (%)**								.59
		Illiterate	12 (10.6)		12 (10.7)				
		Primary school	26 (23)		21 (21.4)				
		Junior school	45 (39.8)		39 (34.8)				
		Senior school	21 (18.6)		31 (27.7)				
		Undergraduate or college	9 (8)		6 (5.4)				
		Postgraduate or above	0 (0)		0 (0)				
	**Employment status, n (%)**								.19
		Employed	11 (9.7)		14 (12.5)				
		Retired	99 (87.6)		98 (87.5)				
		Unemployed	3 (2.7)		0 (0)				
	**Average household monthly income (US $), n (%)**								.38
		<416.10	14 (12.4)		15 (13.4)				
		416.10-832.19	52 (46)		41 (36.6)				
		832.20-1248.30	32 (28.3)		39 (34.8)				
		>1248.30	15 (13.3)		17 (15.2)				
	Duration of diabetes (years), mean (SD)			12.8 (6.3)		13.6 (7.2)		.37	
**Family members**									
	**Gender, n (%)**								.39
		Male	51 (45.1)		57 (50.9)				
		Female	62 (54.8)		55 (49.1)				
	Age (years), mean (SD)			47.9 (14.5)		49.1 (11.8)		.50	
	**Relationship, n (%)**								.61
		Spouse	36 (31.9)		38 (33.9)				
		Son	26 (23)		28 (25)				
		Daughter	7 (6.2)		11 (9.8)				
		Daughter-in-low	35 (31)		31 (27.7)				
		Son-in-low	1 (0.9)		0 (0)				
		Grandson or granddaughter	8 (7.1)		4 (3.6)				
	**Education, n (%)**								.98
		Illiterate	1 (0.9)		1 (0.9)				
		Primary school	12 (10.6)		5 (4.5)				
		Junior school	24 (21.2)		26 (23.2)				
		Senior school	27 (23.9)		39 (34.8)				
		Undergraduate or college	47 (41.6)		37 (33)				
		Postgraduate or above	2 (1.8)		4 (3.6)				
	**Chronic disease, n (%)**								.07
		Yes	70 (61.9)		82 (73.2)				
		No	43 (38.1)		30 (26.8)				

### Effectiveness Outcomes

In the intervention group, HbA_1c_ level (*P*<.001) and nonsupportive behavior (*P*=.03) decreased and the scores for general diet (*P*<.001), specific diet (*P*<.001), exercise (*P*=.002), blood sugar testing (*P*=.02), foot care (*P*<.001), risk knowledge (*P*<.001), personal control (*P*<.001), worry (*P*=.02), optimism bias (*P*=.03), and supportive behaviors (*P*<.001) improved. In the control group, besides general diet (*P*=.002), there were no statistically significant differences between baseline and follow-up (Table 2).

**Table 2:** Primary and secondary effectiveness outcomes. The change in score is the follow-up score minus the baseline score. The 95% CIs that do not contain 0 are statistically used to show the intervention effectiveness after an individual received the intervention. *P* values are the result of a paired-sample *t* test to demonstrate whether the difference was statistically significant.

			Intervention group (n=113)									Control group (n=112)					
			Baseline, mean (SD)		Follow-up, mean (SD)		Change (95% CI)		*P* value		Baseline, mean (SD)		Follow-up, mean (SD)		Change (95% CI)		*P* value
Glycated hemoglobin A_1c_ level (%)			7.90 (0.75)		7.30 (1.07)		–0.60 (–0.82 to –0.38)		<.001		7.84 (0.66)		7.99 (1.22)		0.15 (–0.09 to 0.39)		.22
**Gender (score)**																	
	Male	7.99 (0.87)		7.46 (1.11)		–0.53 (–0.88 to –0.18)		.004		7.80 (0.57)			7.89 (1.29)	0.08 (–0.28 to 0.44)		.66	
	Female	7.83 (0.61)		7.16 (1.01)		–0.62 (–0.95 to –0.37)		<.001		7.87 (0.75)			8.09 (1.14)	0.22 (–0.11 to 0.54)		.18	
**Self–care activities (score)**																	
	General diet	5.36 (2.37)		6.39 (1.09)		1.03 (0.58 to 1.48)		<.001		5.05 (2.36)			5.75 (1.88)	0.70 (0.26 to 1.13)		.002	
	Specific diet	3.59 (1.73)		4.31 (1.36)		0.72 (0.34 to 1.09)		<.001		3.85 (1.96)			3.62 (1.45)	–0.23 (–0.67 to 0.21)		.31	
	Exercise	3.55 (1.96)		4.22 (1.99)		0.67 (0.26, 1.07)		.002		3.83 (1.71)			3.95 (1.97)	0.12 (–0.27 to 0.51)		.55	
	Blood sugar testing	1.53 (1.81)		2.09 (2.07)		0.56 (0.11 to 1.00)		.02		1.77 (2.20)			1.64 (1.66)	–0.13 (–0.51 to 0.26)		.52	
	Foot care	1.56 (2.46)		3.23 (2.76)		1.67 (1.12 to 2.22)		<.001		1.13 (2.17)			1.35 (1.84)	0.22 (–0.29 to 0.75)		.39	
	Smoking	0.79 (0.41)		0.85 (0.36)		0.06 (–0.01 to 0.13)		.07		0.82 (0.39)			0.85 (0.36)	0.03 (–0.02 to 0.07)		.26	
**Risk perception of diabetes (score)**																	
	Risk knowledge	3.32 (1.63)		4.45 (1.13)		1.13 (0.79 to 1.47)		<.001		3.28 (1.43)			3.54 (1.51)	0.26 (–0.08 to 0.60)		.14	
	Personal control	2.94 (0.40)		3.15 (0.44)		0.21 (0.12 to 0.32)		<.001		2.99 (0.38)			2.96 (0.30)	–0.03 (–0.13 to 0.06)		.44	
	Worry	2.73 (0.69)		2.91 (0.55)		0.18 (0.03 to 0.33)		.02		2.74 (0.65)			2.68 (0.57)	–0.06 (–0.20 to 0.08)		.42	
	Optimism bias	2.75 (0.63)		2.92 (0.58)		0.17 (0.01 to 0.32)		.03		2.56 (0.77)			2.66 (0.64)	0.10 (–0.08 to 0.27)		.30	
	Personal risk	2.22 (0.55)		2.11 (0.62)		–0.11 (–0.25, 0.02)		.10		2.22 (0.60)			2.15 (0.59)	–0.07 (–0.22 to 0.05)		.22	
**Family support (score)**																	
	Supportive behaviors	20.25 (6.65)		25.69 (6.67)		5.44 (4.07 to 6.81)		<.001		20.71 (7.11)			20.38 (6.02)	–0.33 (–1.42 to 0.76)		.55	
	Nonsupportive behaviors	29.82 (3.93)		28.71 (4.48)		–1.11 (–2.09 to –0.14)		.03		29.21 (3.30)			28.75 (3.60)	–0.46 (–1.21 to 0.30)		.24	

### The Effect of the eHealth Family-Based Intervention

Patients with T2DM who participated in the eHealth family-based intervention had significantly lower HbA_1c_ values (β=–.69, 95% CI –.99 to –.39; *P*<.001) and improved scores for general diet (β=.60, 95% CI .20-1.00; *P*=.003), special diet (β=.71, 95% CI .34-1.09; *P*<.001), blood sugar testing (β=.50, 95% CI .02-.98; *P*=.04), foot care (β=1.82, 95% CI 1.23-2.42; *P*<.001), risk knowledge (β=.89, 95% CI .55-1.24; *P*<.001), personal control (β=.22, 95% CI .12-.32; *P*<.001), worry (β=.24, 95% CI .10-.39; *P*=.001), optimism bias (β=.26, 95% CI .09-.43; *P*=.003), and supportive behaviors (β=5.52, 95% CI 4.03-7.01; *P*<.001), as shown in Table 3.

**Table 3:** Results of analysis of covariance for the effect of the eHealth family-based intervention. The covariables included patient gender, patient age, patient education, family member education, and family member relationship. We only show covariables with statistical implications.

Effective outcomes			β		SE (95% CI)		*P* value
**Glycated hemoglobin A_1c_ level**							
	Intervention	–.69		.15 (–.99 to –.39)		<.001	
	Baseline hemoglobin A_1c_ level	.26		.11 (.04 to .47)		.02	
**General diet**							
	Intervention	.60		.30 (.20 to 1.00)		.003	
	Baseline general diet	.23		.04 (.14 to .31)		<.001	
**Special diet**							
	Intervention	.71		.19 (.34 to 1.09)		<.001	
**Exercise**							
	Intervention	.38		.25 (–.13 to .88)		.14	
	Baseline exercise	.39		.07 (.24 to .53)		<.001	
**Blood sugar testing**							
	Intervention	.50		.24 (.02 to .98)		.04	
	Baseline blood sugar testing	.33		.06 (.20 to .45)		<.001	
**Foot care**							
	Intervention	1.82		.30 (1.23 to 2.42)		<.001	
	Baseline foot care	.22		.07 (.09 to .35)		.001	
	Patient age	.79		.31 (.17 to 1.41)		.01	
**Smoking**							
	Intervention	.02		.04 (–.05 to .09)		.56	
	Baseline smoking	.57		.05 (.47 to .67)		<.001	
	Patient gender	.09		.04 (.01 to .17)		.03	
**Risk knowledge**							
	Intervention	.89		.18 (.55 to 1.24)		<.001	
	Baseline risk knowledge	.15		.06 (.03 to .26)		.02	
	Patient age	.49		.18 (.12 to .89)		.009	
**Personal control**							
	Intervention	.22		.05 (.12 to .32)		<.001	
**Worry**							
	Intervention	.24		.08 (.10 to .39)		.001	
	Baseline worry	.15		.06 (.04 to .26)		.009	
**Optimism bias**							
	Intervention	.26		.09 (.09 to .43)		.003	
**Personal risk**							
	Intervention	–.02		.08 (–.17 to .13)		.81	
	Baseline personal risk	.28		.07 (.14 to .41)		<.001	
	Patient age	.23		.08 (.08 to .38)		.004	
	Family member education	.10		.04 (.02 to .17)		.01	
	Family member relationship	–.05		.02 (–.10 to –.004)		.03	
**Supportive behaviors**							
	Intervention	5.52		.75 (4.03 to 7.01)		<.001	
	Baseline supportive behaviors	.47		.06 (.36 to .58)		<.001	
**Nonsupportive behaviors**							
	Intervention	–.25		.54 (–1.32 to .82)		.65	
	Baseline nonsupportive behaviors	.28		.07 (.13 to .43)		<.001	

## Discussion

### Principal Findings

The study was a single-center, 2-parallel-group randomized controlled trial to assess the effectiveness of an eHealth family-based intervention. This structured intervention program assessed knowledge, attitude, and behaviors. The difference between the intervention and control groups was whether the family members of the patients with T2DM followed the official WeChat account. This study confirmed that this intervention could enhance glucose control, self-care activities, and risk perception among patients with T2DM. Additionally, the patients were better able communicate with community health service providers and obtain knowledge of diabetes-related risks, benefits, and skills after their family members reminded them of inappropriate self-management behaviors and inadequate risk perception of T2DM. Family support of the patients was also improved.

### Comparison With Prior Work

Our principal findings are similar to those of McEwen et al [50], Cai and Hu [51], and Wichit et al [52], who implemented interventions via health education classes, home visits, and telephone calls. These interventions can be classified as in-person [53], a type of delivery that may not only waste a large amount of staff resources and time but may also lack sustainability due to poor compliance. During the intervention process, the intervention tool of the studies mentioned above was a booklet. Although participants received health education, the education model was only on-site, and the education content was the same as that in the booklet. Additionally, telephone-based interventions were completed by telephone. Hemmati Maslakpak et al [54] reported holding on-site health education classes and contacting participants who needed to attend classes at a fixed time. Compared with the characteristics of the aforementioned studies, this study implemented an eHealth family-based intervention via an official WeChat account without time or site limitations. Family members of patients could read the online intervention articles at any time, and the offline intervention only focused on skills practice, in order to enable the family members to complete the tasks by themselves in daily life.

There is another principal finding worth noting: although the eHealth family-based intervention was effective, patients with T2DM who participated in the intervention did not achieve ideal glucose control (HbA_1c_ <7%). This finding is similar to that of a single-arm study of an eHealth intervention program that used an accessible app (Vida Health) as the intervention platform, which is similar to WeChat [55]. The inability of patients with T2DM to achieve ideal control could be explained by the fact that only patients with uncontrolled T2DM (HbA_1c_ >7%) were enrolled in our study. Glycemic control in patients with T2DM is complex and is influenced by various factors, such as medicine adjustment, regular visits to a doctor, and lifestyle; the presence of these factors was confirmed during interviews with doctors and community health service providers. While ideal control was not achieved, patients with T2DM in the intervention group still had significantly lower HbA_1c_ levels than patients in the control group after 12 months, suggesting that the eHealth family-based intervention was an effective way to improve glucose control. In future studies, specific factors influencing glucose control should be considered in combination with an eHealth family-based intervention to educate patients with T2DM and their family members.

Sun et al [19] conducted a study that was similar to ours based on a diet intervention conducted by dietitians among outpatients; their intervention was more effective (HbA_1c_ decreased from 7.84% to 6.84%) than ours (HbA_1c_ decreased from 7.9% to 7.3%). A possible reason is that the health providers were from a tertiary hospital and could provide more professional suggestions, making the patients more willing to comply. However, dietitian-based interventions are difficult to generalize due to the shortage of these professionals. Our study was a community-based intervention, and the community was a national health-management platform for patients with T2DM. Therefore, our community-based intervention is more generalizable in real life.

Yang et al [56] also conducted a study of patients with T2DM and obtained good results; however, effectiveness in their study (a change in HbA_1c_ of 0.3%) was lower than in our study. One possible reason is that their intervention content was based only on the medical guidelines of the Korean Diabetes Association, while our study developed intervention content based on the National Standard for Basic Public Health Services [14] and provided specific family support. Although the Guidelines for the Prevention and Treatment of Type 2 Diabetes Mellitus in China (2020 revision) [3] reported that family members also play an important role in health management for patients with T2DM, this guideline does not provide specific measures. Our study added a specific family member–based health intervention to the basic health intervention in accordance with the guideline. Another possible reason for the differences between this study and the previous one is that we added information on risk perception during the process of health intervention development, which could have improved our health intervention.

New eHealth interventions, with their improved accessibility, have the potential to replace in-person interventions [57]. Despite their potential benefits, we identified certain limitations of eHealth interventions in our study. First, social support can be classified into 4 types: emotional, instrumental, informational, and appraisal [53,58,59]. It is, however, difficult to evaluate how eHealth interventions may contribute to the social support of patients with T2DM [53]. Second, commonly used eHealth interventions, such as text messages, apps, and web-based programs, are known to have a positive impact on the self-management behaviors of patients with T2DM; however, due to limitations related to character count per message, content type, interactivity, cost, accessibility, and internet connection capacity, it is difficult to compare and generalize findings across studies [60].

Considering the aforementioned limitations of eHealth interventions in previous studies, we used WeChat, a common and free app used daily by Chinese people [24,25], to address limitations related to accessibility, cost, and internet connection capacity, although we did not consider instrument support in our study. Additionally, for the delivery of the articles, we classified them into 3 types and delivered them in different ways, which solved the limitation related to character count. For articles in the official WeChat account, different types of content, such as video content, could be linked at the bottom of each article, which solved the limitation related to content type. To evaluate the extent of social support in our study, we sufficiently considered the impact of family members on patients with T2DM when designing the intervention tools by referring to the KAP and HBM conceptual frameworks.

### Strengths and Limitations

The major strength of this study is that the eHealth family-based intervention used content usually delivered as part of in-person health education, as set out in the National Standard for Basic Public Health Services. The dropout rate of participants was very low (only 3 pairs of participants).

However, this study also has some limitations. First, the sample was small and only included patients enrolled in 1 city. Future studies should include larger samples from multiple sources. Second, the intervention time was 1 year, and we did not perform continuous follow-up of the patients with T2DM and their family members. Consequently, we could only conclude whether the intervention was effective over a period of time; we could not confirm its long-term effects. Third, participant compliance was not comprehensively documented; for example, we did not determine how long or how often patients with T2DM or their family members read the intervention articles. Fourth, due to a reduced workforce and funding restrictions, we did not collect data at 6 months. Restrictions also prevented us from collecting some indicators that were included in the protocol, such as weight, height, waist circumference, hip circumference, blood pressure, and BMI.

Additionally, during the process of intervention implementation, we revised the inclusion and exclusion criteria that were published in the protocol to remove “no history of diabetes” for the family members and add “never participated in other, similar research.” We made this revision because the study aimed to find an intervention program that is suitable for the real world. After the protocol was developed, we came to understand that the family members of patients with T2DM can also develop T2DM. If we had recruited participants based on the inclusion and exclusion criteria in the protocol, a large number of suitable participants would have been lost, because T2DM is a common disease in older adults. The patients with T2DM were often older people whose family members were a spouse, their child, or a son- or daughter-in-law; the mean age of these family members was greater than 45 years. In addition, to avoid influence from other research, we required that the family members had not participated in other research that was similar to our study.

### Conclusions

We conducted a structured eHealth family-based intervention program that included T2DM-related knowledge, risks, and skills to enhance the ability and risk perceptions of family members regarding T2DM. This intervention was able to effectively help patients with T2DM to improve their glucose control by promoting participation in community health management and strengthening their self-management behaviors. Overall, this eHealth family-based intervention for patients with uncontrolled T2DM is a promising way to empower family members to support these patients in their endeavors to improve self-management behaviors. Our study also provides information for community health providers to develop health intervention content and mobile health intervention platforms for patients with uncontrolled T2DM.

## Appendix

Multimedia Appendix 1 The list of intervention articles’ themes.

Multimedia Appendix 2 Online intervention flowchart.

Multimedia Appendix 3 The meanings of each indicator in Risk Perception Survey-Diabetes Mellitus.

Multimedia Appendix 4 CONSORT-eHEALTH checklist (V 1.6.1).

## Abbreviations

CONSORT: Consolidated Standards of Reporting Trials
HbA_1c_: glycated hemoglobin A_1c_
HBM: Health Belief Model
KAP: Knowledge-Attitude-Practice
T2DM: type 2 diabetes mellitus
