# Supplementary material for: The Effectiveness of an eHealth Family-Based Intervention Program in Patients With Uncontrolled Type 2 Diabetes Mellitus (T2DM) in the Community Via WeChat: Randomized Controlled Trial
Source: JMIR Mhealth Uhealth. 2023 Mar 20;11:e40420. doi: 10.2196/40420 (PMC10131610; doi:10.2196/40420)
Supplement: Multimedia Appendix 1 [file mhealth_v11i1e40420_app1.docx]

Table 1 The list of intervention articles’ themes

| **Basic knowledge** | **Skills** | **Risk knowledge** |
| --- | --- | --- |
| 1. Correct understanding of blood glucose and insulin  2. Definition, symptoms and diagnostic criteria of diabetes  3. Diabetes comprehensive control objectives and hospital testing items  4. Medication mistakes  5. Different types of oral hypoglycemic drugs and their time  6. Precautions for taking common drugs  7. Insulin (Common misconception)  8. Main principles of diet management  9. Dietary management precautions  10. The Benefits of exercise and the disadvantages of not exercising  11. Contraindications to exercise  12. Content and standard of blood glucose monitoring  13. Significance of blood glucose monitoring  14. Precautions for blood glucose monitoring  15. Blood pressure and lipid management in diabetic patients | 1. Special case handling skills  2. Insulin injection specifications  3. Precautions for insulin injection  4. How do diabetic patients calculate the total calories needed in a day  5. The palm rule  6. Food in exchange  7. Diet control skills  8. Frequency, timing and intensity of exercise  9. Comparison of common sports  10. Related auxiliary activities before, during and after exercise  11. Selection and recording of blood glucose monitoring frequency  12. Blood glucose monitoring procedure | 1. Brief introduction of hypoglycemia (inducements, symptoms, etc.)  2. Prevention and treatment of hypoglycemia in diabetic patients  3. Brief introduction of diabetic ketoacidosis (inducement, symptoms, etc.)  4. Prevention and treatment of diabetic ketoacidosis  5. Brief introduction of hyperglycemia and Hyperosmolality syndrome (inducement, symptoms, etc.)  6. Prevention and treatment of hyperglycemia and hyperosmolality syndrome  7. Brief introduction of diabetic lactic acid poisoning (inducement, symptoms, etc.)  8. Prevention and treatment of lactic acid poisoning  9. Brief description of diabetic kidney disease (inducement, symptoms, etc.)  10. Prevention and treatment of diabetic nephropathy  11. Brief Introduction of diabetic retina (causes, symptoms, etc.)  12. Prevention and treatment of diabetic retinopathy  13. Diabetic neuropathy (triggers, symptoms, etc.)  14. Prevention and treatment of diabetic neuropathy  15. Diabetic cardiovascular and cerebrovascular diseases (inducements, symptoms, etc.)  16. Prevention and treatment of diabetic cardiovascular and cerebrovascular diseases  17. Lower extremity vascular disease (inducement, symptoms, etc.)  18. Prevention and treatment of lower extremity vascular diseases  19. Diabetic foot disease (inducement, symptoms, etc.)  20. Prevention and treatment of diabetic foot (pulsatile examination of dorsal foot artery, daily care, screening, treatment)  21. Other diseases and diabetes |
