# Supplementary figures and images for: The Effectiveness of an eHealth Family-Based Intervention Program in Patients With Uncontrolled Type 2 Diabetes Mellitus (T2DM) in the Community Via WeChat: Randomized Controlled Trial
Source: JMIR Mhealth Uhealth. 2023 Mar 20;11:e40420. doi: 10.2196/40420 (PMC10131610; doi:10.2196/40420)

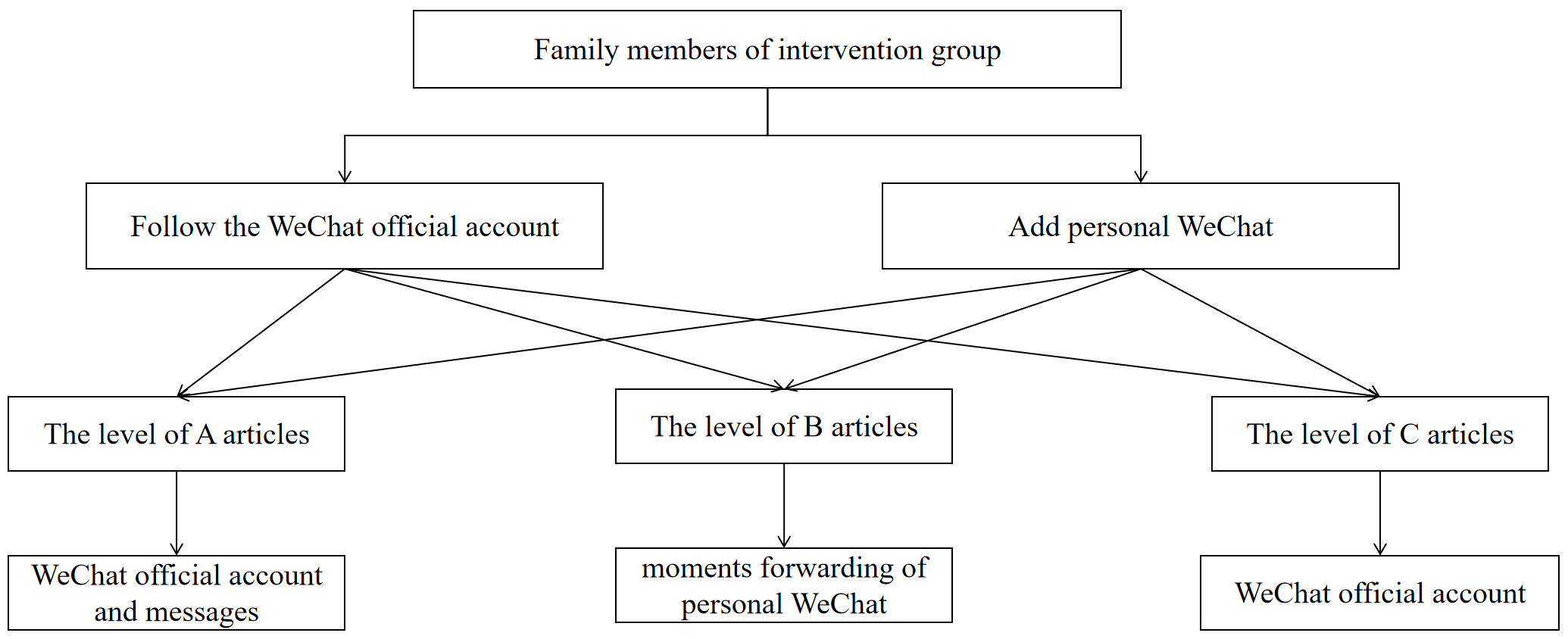


Figure 1 Online intervention flowchart

Supplement: Multimedia Appendix 2 [file mhealth_v11i1e40420_app2.docx]
