# Supplementary material for: The Effectiveness of an eHealth Family-Based Intervention Program in Patients With Uncontrolled Type 2 Diabetes Mellitus (T2DM) in the Community Via WeChat: Randomized Controlled Trial
Source: JMIR Mhealth Uhealth. 2023 Mar 20;11:e40420. doi: 10.2196/40420 (PMC10131610; doi:10.2196/40420)
Supplement: Multimedia Appendix 3 [file mhealth_v11i1e40420_app3.docx]

Table 1 The meanings of each indicators in Risk Perception Survey-Diabetes Mellitus

| Indicators | Meanings and implications |
| --- | --- |
| Risk knowledge | The acquirement of type 2 diabetes mellitus related knowledge if patients have poor glucose control level. The higher score is better. |
| Personal control | The ability of type 2 diabetes mellitus patients taking measures to prevent them to suffer from bad health outcomes. The higher score is better. |
| Worry | The feeling of type 2 diabetes mellitus patients who may suffer from bad health outcomes if they have poor glucose control level. The higher score is better, which indicates that T2DM patients worry about their health status much more. |
| Optimism bias | The feeling of type 2 diabetes mellitus patients who realize that they have good health outcomes if they are at good glucose control level. The higher score is better, which indicates that T2DM patients feel more optimistic that they can achieve good health status if they do their utmost to control their glucose. |
| Personal risk | The feeling of type 2 diabetes mellitus patients who may develop complications if they have poor glucose control. The higher score is better, which indicates that T2DM patients feel more risk to develop complications if they do not control glucose control well. |
